# Association between use of ß2-adrenergic receptor agonists and incidence of Parkinson’s disease: Retrospective cohort analysis

**DOI:** 10.1371/journal.pone.0276368

**Published:** 2022-11-28

**Authors:** Hasan Nadeem, Bo Zhou, Dana Goldman, John Romley

**Affiliations:** 1 Department of Medicine, University of Washington, Seattle, Washington, United States of America; 2 Schaeffer Center for Health Policy and Economics, University of Southern California, Los Angeles, California, United States of America; 3 School of Pharmacy, University of Southern California, Los Angeles, California, United States of America; 4 Price School of Public Policy, University of Southern California, Los Angeles, California, United States of America; Gulu University, UGANDA

## Abstract

**Introduction:**

Previous observational studies assessing β2-agonist/-antagonist use on PD risk have yielded conflicting results. We evaluated the relationship between β2-agonist use and the incidence of Parkinson’s disease in patients with chronic lung disease.

**Methods:**

We performed a retrospective cohort analysis on a 20% random sample abstracted from a traditional (fee-for-service) Medicare program in the United States. Inclusion criteria were individuals over 65 years old diagnosed with asthma, COPD, and/or bronchiectasis who were enrolled in a prescription drug (standalone Part D) plan over 2007–2010 and alive through 2014. The main outcome measure was a diagnosis of Parkinson’s disease over the period 2011–2014, in relation to the number of 30-day-equivalent drug claims over 2007–2010. Logistic regression analysis was performed on a sample including 236,201 Medicare beneficiaries.

**Results:**

The sample was 68% female, 80% white, and on average 77 years old as of 2010. Compared to non-users, β2-agonist users were more likely to be younger (76.3y versus 78.0y), smokers (40.4% versus 31.1%) and asthmatic (62.4% versus 28.3%). The odds ratio for a β2-agonist claim on PD development was 0.986 (95% CI 0.977–0.995) after adjusting for demographics, smoking history, respiratory exacerbations, comorbidities, and other drug use. Risk reductions were larger for males than females (0.974 versus 0.994, *P* = 0.032), and for individuals with COPD compared to those with asthma (0.968 versus 0.998, *P* = 0.049). Reverse causality was addressed with a Cox analysis that allowed β2-agonist use to vary from medication initiation to disease onset. By the end of the follow-up period, β2-agonist use was shown to be associated with a true protective effect against PD onset.

**Discussion:**

β2-agonist use is associated with decreased risk of PD incidence. Further investigation, possibly including clinical trials, is warranted to strengthen the evidence base supporting clinical decision-makers looking to repurpose pharmaceuticals to prevent neurodegenerative disease onset.

## Introduction

Parkinson’s disease (PD) is the second most common neurodegenerative disorder in the United States. The prevalence of PD is about 1% in individuals over the age of 60 [1]. It is projected that by 2040, the number of people living with PD in the United States will be around 770,000 individuals at minimum [2]. The economic cost incurred by Parkinson’s disease in 2010 was over $14.4 billion USD [3]. In the United States, an increasing life expectancy and elderly population are poised to dramatically increase the societal burden of Parkinson’s disease.

Parkinson’s disease is a movement disorder clinically diagnosed by the classic triad: tremors, rigidity, and bradykinesia [4]. However, PD broadly affects the nervous system and it is widely recognized that the disease has diverse symptomology that includes several non-motor manifestations [5, 6]. The clinical manifestations of Parkinson’s disease are thought, in part, to be due to a resultant dopamine deficiency and distinctive neuropathologic features. On autopsy, the brains of individuals with Parkinson’s disease show a characteristic degeneration of dopaminergic neurons in the substantia nigra and an accumulation of the α-synuclein protein, which forms Lewy bodies [7].

Approximately 90% of PD cases occur sporadically and the cause of disease onset is not yet known. As such, the degree to which dysregulated α-synuclein production affects PD pathogenesis is unclear and remains the subject of further study. Various cellular and animal models have shown that overexpression of α-synuclein leads to neuronal death and neurodegeneration [8–10]. Genetic studies on monogenic forms of PD (less than 10% of cases) have shown that point mutations in the *SNCA* gene, which encodes for the α-synuclein protein, to be a cause of disease onset [11]. Additional studies have also shown that common copy number variants in the *SNCA* gene are also associated with sporadic PD [12]. While not all causes of Parkinson’s disease are due to a mutated *SNCA* gene, these findings show that genetics and heritability, particularly as it relates to the *SNCA* gene, play a role in many individuals’ development of Parkinson’s disease.

In a recent study conducted by Mittal *et*. *al*. [13], it was shown that β2-adrenergic receptors (β2AR) are bidirectional regulators of *SNCA* expression. In this study, experimental models were used to show that β2-agonists and antagonists were shown to decrease and increase neuronal *SNCA* expression, respectively. The authors conducted an epidemiological study using nationwide pharmacy data in a Norwegian population, which reinforced the findings in the experimental models. In this longitudinal analysis, the use of the β2-agonist albuterol was associated with a lower risk of PD development. Conversely, use of the beta antagonist propranolol was associated with a higher risk of developing PD [13].

A study conducted by Nielsen *et*. *al* more closely evaluated the longitudinal associations between beta-agonists and antagonists and Parkinson’s disease risk [14]. This group used a case-control study design and concluded that after adjusting for tremor and lagging β2AR antagonist exposure, the increased risk for PD dissipated. Likewise, after adjusting for smoking, the decreased risk of PD in β2-agonist users was close to null. Overall, the group concluded that confounding factors were largely responsible for differences in PD risk in the Norwegian epidemiological study [14]. Hopfner *et al*. corroborated these results with a case-control study utilizing Danish health registry data, finding the association with β2-agonist use and PD risk to be indirectly mediated through smoking [15].

The contradictory results between the aforementioned studies yields an inconclusive understanding of the effect of β2AR modulation on PD risk at the population level. Despite an improvement in controlling for potential confounding factors in the latter study, there are areas for improvement. Firstly, it is imperative to evaluate the effect of β2AR medications in a diverse setting that allows researchers to assess the effects of demographic variations. Additionally, it is important to adjust for other risk factors alongside smoking exposure that may impact PD onset, such as asthma severity [16].

In our study, we aimed to evaluate the association between β2AR use and PD incidence while addressing several concerns in the literature. We used administrative data from a 20% random sample of Medicare enrollees from 2007 to 2014 and identified patients with asthma, chronic obstructive pulmonary disease (COPD), and/or bronchiectasis, and later PD, with corresponding diagnostic codes. We designated the exposure period to be between 2007–2010 and set the following four years (2011–2014) as the outcome period. We performed logistic regression analyses on the onset of Parkinson’s disease in β2-agonist users and non-users with asthma/COPD, while controlling for tobacco use, other medication use, comorbidities, age, sex, and race. Finally, we addressed the possibility of reverse causality with a Cox (Proportional Hazard) analysis that assessed variations in β2-agonist use between time of medication initiation and PD onset.

## Methods

The study was approved by the University Park Institutional Review Board at the University of Southern California (UP-20-00085). Given the use of de-identified claims data, consent was not required for this study.

### Study design

We performed a retrospective cohort analysis on a 20% random sample of Medicare beneficiaries from 2007–2014. Individuals were selected into the sample if they were over the age of 65 and had a diagnosis of asthma, COPD, and/or bronchiectasis by 2007 and no diagnosis of Parkinson’s disease prior to 2010. Beneficiaries were grouped as β2-agonist users or non-users. We aggregated total β2-agonist drug use from Parts A, B, and D using NDCs (National Drug Codes) selected from First Data Bank Drug database and HCPCS (Healthcare Common Procedure Coding System) J and Q codes derived from the Centers for Medicare and Medicaid Services NDC-HCPCS crosswalk tables. Β2-agonist exposure is measured by total 30-day equivalent claims derived from total HCPCS units supplied in Parts A and B and total days supplied in part D. In the outcome period, 2010–2014, we measured onset of Parkinson’s disease within our sample.

### Study sample

The study population is composed of a 20% random sample of Medicare beneficiaries from 2007–2014 that have a diagnosis of asthma, COPD, and/or bronchiectasis. Β2-agonists are used as bronchodilators and comprise the frontline maintenance therapy for asthma and COPD [17]. Individuals in the sample were enrolled in FFS part A/B and lived through 2014, and enrolled in standalone Part D for all months between 2007–2010. We used validated algorithms from CCW (Chronic Conditions Warehouse) and ICD-9 (*International Classification of Diseases*, *Ninth Revision*, *Clinical Modification*) codes to backdate the health histories of individuals enrolled in fee-for-service (FFS) Part A/B health histories from 2002.

We used the ICD-9 codes 493.0x – 493.2x and 493.8x – 493.9x to identify individuals with asthma, 494.0 to identify individuals with bronchiectasis, and 490.xx– 492.xx and 496.xx to identify individuals with COPD. A diagnosis of Parkinson’s disease onset was identified with any claim with the ICD-9 code 332.0. Individuals diagnosed with essential tremor (ICD-9 code 333.1) were removed from the sample to exclude potential misdiagnoses of Parkinson’s disease [18].

### Statistical analysis

In our main analysis, we estimated a multivariable logistic regression to evaluate the relationship between β2-agonist use and Parkinson’s disease. The outcome variable was the four-year incidence of PD, as measured by a claim during the outcome period. The primary exposure variable was any 30-day claim for β2-agonist medications. We adjusted for patient demographics and comorbidities through the end of 2010. It has been shown that tobacco exposure and asthma severity are associated with a decreased and an increased risk of developing Parkinson’s disease, respectively [16, 19]. As such, we controlled for tobacco exposure by identifying presence of smoking history with smoking cessation claims from Medicare Parts A/B and smoking deterrents in Part D through 2010 [20]. We controlled for asthma / COPD severity using the number of inpatient skilled nursing facility (SNF) stays with corresponding ICD-9 codes for asthma or COPD exacerbation. We also adjusted for the use of other asthma medications as well as prescription drugs that may affect onset of Parkinson’s disease, such as: leukotriene modifiers [21], glucocorticoids [22], anticholinergics [23], beta-blockers [13], NSAIDs [24], and statins [25].

The literature has raised concerns about the possibility of reverse causality, in which early symptoms of undiagnosed PD may influence β2-agonist use [15, 26]. For example, early presentation of Parkinson’s disease can cause respiratory dysfunction in the form of dyspnea, upper airway obstruction, or an impaired cough reflex, which may prompt an individual with chronic lung disease to utilize additional beta-agonists before a true diagnosis of PD is made [27]. We addressed these concerns by performing a Cox analysis on time of β2-agonist initiation to Parkinson’s disease onset in which the relationship with β2-agonist use could vary over time [28, 29]. To do so, we included β2-agonist use interacted with time (measured in months) as a covariate, in addition to β2-agonist use on its own (and all other covariates from the preceding model). In this Cox analysis an elevated risk of Parkinson’s onset shortly after β2-agonist initiation would be consistent with reverse causality, whereas a decreased risk of PD onset by the end of follow-up period would be consistent with a true protective effect. All statistical analysis was completed using Stata, version 16.

## Results

Our sample included 236,201 beneficiaries. The demographic characterization of our sample is 68% female, 80% white, and on average 77 years old at the beginning of 2010 (Table 1). At the beginning of the exposure period, 85% (191,574) of the sample had a diagnosis COPD or bronchiectasis and 47% (99,467) had a diagnosis of asthma. Severe asthma was found in 2.9% (6,953) of beneficiaries, which was defined as having one or more inpatient or SNF stays with an asthma exacerbation between 2007–2010. Severe COPD was found in 5.7% (13,425) of beneficiaries, which was defined similarly to severe asthma with one or more inpatient or SNF stays with a COPD exacerbation between 2007–2010. The average number of inpatient or skilled nursing facility stays for patients with exacerbations from severe asthma or COPD were 1.4 and 1.6, respectively. More than a third (36.1%) had a history of smoking.

**Table 1:** Descriptive statistics of study population.

Characteristics	Full Sample (N = 236,201)
Incident Parkinson’s Disease Diagnosis (%)	1.87
Any β2-Agonist Use (%)	53.7
Age, Mean (SD), Years	77.1 (6.1)
Male (%)	32.1
Race, White (%)	80.1
Race, Hispanic (%)	7.81
Race, Black (%)	6. 82
Race, Asian (%)	3.84
Race, Other (%)	1.39
Ever Smoker (%)	36.1
Asthma Exacerbations (%)	4.84
COPD Exacerbations (%)	10.2
Comorbidities (%)	
Acute myocardial infarction	6.60
Anemia	66.6
Asthma	46.7
Atrial fibrillation	18.1
Benign Prostatic Hyperplasia	18.5
Cancer, Breast	6.62
Cancer, Colorectal	3.79
Cancer, Endometrial	1.03
Cancer, Lung	2.31
Cancer, Prostate	5.23
Cataracts	81.9
Chronic Kidney Disease	25.3
Chronic Obstructive Pulmonary Disease	84.6
Congestive Heart Failure	42.5
Alzheimer’s Disease and Related Disorders or Senile Dementias, Other	15.3
Depression	36.8
Diabetes Mellitus	43.6
Glaucoma	28.0
Hip fracture	4.38
Hyperlipidemia	86.5
Hypertension	91.0
Hypothyroidism	26.9
Ischemic Heart Disease	67.6
Osteoporosis	33.9
Rheumatoid Arthritis/Osteoarthritis	71.0
Stroke/Transient Ischemic Attack	19.7
Other drug use (%)	
ACE inhibitors	46.3
Anticholinergics	18.0
ARBs	30.0
Beta Blocker (Any, Selective)	41.8
Beta Blocker (Any, Non-Selective)	12.8
Ca^2+^ Channel Blockers	5.94
COX-2 Inhibitors	10.1
Glucocorticoids	49.3
Leukotriene Modifiers	14.6
Loop Diuretics	30.6
NSAIDs	42.7
Statins	60.9
Thiazide Diuretics	36.2
Xanthine Derivatives	5.45

Notes: Age was measured as of 2010. SDs are in parentheses.

Table 2 shows descriptive statistics by β2-agonist use. Users constituted 53.7% (126,882) of the sample and had on average four 30-day equivalent claims per year (standard deviation = 5.1). Among β2-agonist users, 94.1% filled Part D claims and 32.2% had a β2-agonist prescription from Part A/B as indicated by HCPCS codes. The group of β2-agonist users, on average, were more likely to be younger (73.3 years vs 75.0 years), male (69.6% vs 65.9%), smokers (40.4% vs 31.1%), and asthmatic (62.4% vs 28.3%). There were 90% of beneficiaries who used short-acting β2-agonists and 52% of beneficiaries who used long-acting β2-agonists. Albuterol sulfate (99% of short-acting claims) and salmeterol (76% of long-acting claims) were the most common short-acting and long-acting β2-agonists, respectively. From 2011 through 2014, 1.87% (4,413) individuals in our study were diagnosed with Parkinson’s disease. The unadjusted 4-year incidence rate among β2-agonist users did not differ from the rate for non-users (1.86% vs 1.88%, P = 0.70).

**Table 2:** Descriptive statistics by β2-agonist use.

Characteristic	No Use (N = 109,319)	Use (N = 126,882)	P value
Incident Parkinson’s Disease Diagnosis (%)	1.88	1.86	0.70
Age, Mean (SD), Years	78.0	76.3	<0.001
Male (%)	34. 0	30.4	<0.001
Race, White (%)	81.3	79.2	<0.001
Race, Hispanic (%)	7. 40	8.39	<0.001
Race, Black (%)	6.45	7.13	<0.001
Race, Asian (%)	3.95	3.75	0.014
Race, Other (%)	1.19	1.56	<0.001
Ever Smoker (%)	31.1	40.4	<0.001
Asthma Exacerbations (%)	0.33	5.20	<0.001
COPD Exacerbations (%)	1.04	9.68	<0.001
Comorbidities (%):			
Acute myocardial infarction	6.78	6.45	0.002
Alzheimer’s Disease and Related Disorders or Senile Dementia	15.8	14.8	<0.001
Anemia	67.7	65.7	<0.001
Asthma	28.3	62.4	<0.001
Atrial fibrillation	18.8	17.5	<0.001
Benign Prostatic Hyperplasia	19.9	17.4	<0.001
Cancer, Breast	6.84	6.43	<0.001
Cancer, Colorectal	4.25	3.40	<0.001
Cancer, Endometrial	1.04	1.03	0.79
Cancer, Lung	1.77	2.77	<0.001
Cancer, Prostate	5.81	4.74	<0.001
Cataracts	82.4	81.5	<0.001
Chronic Kidney Disease	24.7	25.8	<0.001
Chronic Obstructive Pulmonary Disease	85.2	84.1	<0.001
Congestive Heart Failure	39.8	44.8	<0.001
Depression	33.9	39.4	<0.001
Diabetes Mellitus	42.6	44.5	<0.001
Glaucoma	28.4	27.5	<0.001
Hip fracture	4.93	3.90	<0.001
Hyperlipidemia	87.0	86.1	<0.001
Hypertension	91.0	91.0	0.88
Hypothyroidism	26.7	27.1	0.017
Ischemic Heart Disease	68.0	67.2	<0.001
Osteoporosis	34.0	33.8	0.28
Rheumatoid arthritis	71.1	70.8	0.15
Stroke/Transient Ischemic Attack	20.4	19.1	<0.001
Other drug use (%):			
ACE inhibitors	45.3	47.1	<0.001
Anticholinergics	5.47	28.9	<0.001
ARBs	28.3	31.5	<0.001
Beta Blocker (Any, Selective)	43.5	40.4	<0.001
Beta Blocker (Any, Non-Selective)	12.6	12.9	0.053
Ca^2+^ Channel Blockers	6.0	5.93	0.83
COX-2 Inhibitors	9.3	10.8	<0.001
Glucocorticoids	21.9	73.0	<0.001
Leukotriene Modifiers	5.1	22.9	<0.001
Loop Diuretics	25.9	34.6	<0.001
NSAIDs	39.6	45.4	<0.001
Statins	59.8	61.9	<0.001
Thiazide Diuretics	34.7	37.6	<0.001
Xanthine Derivatives	3.23	7.36	<0.001

Notes: Age was measured as of 2010. SDs are in parentheses.

Our logistic regressions account for a dose-dependent effect of medication usage by measuring the number of β2-agonist 30-day-claims. Without adjustment (specification 1 of Table 3), the odds ratio (OR) for β2-agonist use was 0.987 (95% CI, 0.980–0.995; P = 0.0008). The odds ratios were similar when adjusting for demographics (specification 2) (OR = 0.992; 95% CI, 0.984–0.999; P = 0.024) and comorbidities (specification 3) (OR = 0.990; 95% CI, 0.982–0.997; P = 0.009).

**Table 3:** Multivariate logistic regression of Parkinson’s disease incidence on β2-agonist use (30-day claims).

	(1)	(2)	(3)	(4)
	Unadjusted	Demographics	(2) + comorbidities	(3) + smoking, severity, drugs
*Variable*	*Odds ratio (95% CI)*			
Constant	0.0195*** (0.019–0.020)	4.83e-08*** (5.5^−10^–4.2^−6^)	2.53e-07*** (2.7^−9^–2.4^−5^)	1.89E-07*** (2.0^−9^–1.8^−5^)
# of 30-day claims	0.987*** (0.980–0.995)	0.992** (0.984–0.999)	0.990*** (0.982–0.997)	0.986*** (0.977–0.995)
Age		1.352*** (1.207–1.514)	1.286*** (1.145–1.443)	1.296*** (1.154–1.455)
Male		1.457*** (1.370–1.550)	1.612*** (1.458–1.783)	1.646*** (1.488–1.822)
Race, Hispanic		1.305*** (1.180–1.443)	0.961 (0.866–1.066)	0.946 (0.852–1.052)
Race, Black		0.876** (0.768–0.998)	0.738*** (0.645–0.845)	0.742*** (0.648–0.850)
Race, Asian		1.230*** (1.070–1.414)	1.121 (0.971–1.294)	1.075 (0.929–1.244)
Race, Other		1.184 (0.927–1.512)	1.093 (0.854–1.400)	1.076 (0.840–1.379)
*Comorbidities*: Acute myocardial infarction			0.758*** (0.668–0.860)	0.768*** (0.676–0.873)
Alzheimer’s Disease and Related Disorders or Senile Dementia			2.120*** (1.973–2.279)	2.115*** (1.967–2.273)
Anemia			1.161*** (1.075–1.253)	1.151*** (1.066–1.243)
Asthma			1.106*** (1.032–1.186)	1.073* (0.998–1.154)
Atrial fibrillation			0.993 (0.918–1.073)	0.986 (0.911–1.067)
Benign Prostatic Hyperplasia			1.168*** (1.055–1.292)	1.159*** (1.047–1.282)
Cancer, Breast			1.016 (0.895–1.154)	1.018 (0.896–1.156)
Cancer, Colorectal			1.060 (0.917–1.225)	1.063 (0.920–1.229)
Cancer, Endometrial			0.885 (0.640–1.223)	0.883 (0.638–1.220)
Cancer, Lung			0.944 (0.770–1.159)	0.963 (0.784–1.182)
Cancer, Prostate			0.928 (0.816–1.057)	0.925 (0.812–1.053)
Cataracts			1.127*** (1.033–1.230)	1.121** (1.027–1.223)
Chronic Kidney Disease			1.017 (0.950–1.090)	1.023 (0.954–1.098)
Chronic Obstructive Pulmonary Disease			1.001 (0.907–1.106)	1.005 (0.908–1.112)
Congestive Heart Failure			1.057 (0.987–1.132)	1.029 (0.956–1.107)
Depression			1.571*** (1.472–1.676)	1.562*** (1.464–1.668)
Diabetes Mellitus			1.171*** (1.097–1.249)	1.166*** (1.092–1.246)
Glaucoma			0.998 (0.933–1.066)	0.995 (0.931–1.064)
Hip fracture			1.070 (0.939–1.218)	1.074 (0.943–1.223)
Hyperlipidemia			0.952 (0.862–1.050)	0.964 (0.867–1.071)
Hypertension			0.959 (0.844–1.090)	1.010 (0.885–1.153)
Hypothyroidism			1.022 (0.954–1.095)	1.013 (0.945–1.086)
Ischemic Heart Disease			1.244*** (1.148–1.349)	1.249*** (1.151–1.355)
Osteoporosis			1.103*** (1.028–1.183)	1.096** (1.021–1.176)
Rheumatoid arthritis			1.214*** (1.124–1.311)	1.172*** (1.083–1.269)
Stroke/Transient Ischemic Attack			1.207*** (1.125–1.295)	1.218*** (1.135–1.307)
Ever Smoker				0.900*** (0.841–0.963)
Asthma Exacerbations				0.988 (0.910–1.073)
COPD Exacerbations				1.023 (0.972–1.076)
*Other Drug Use (%)*: ACE inhibitors				0.918** (0.859–0.982)
Anticholinergics				0.999 (0.918–1.087)
ARBs				0.939* (0.872–1.010)
Beta Blocker (Any, Selective)				0.981 (0.920–1.047)
Beta Blocker (Any, Non-Selective)				1.018 (0.929–1.114)
Ca^2+^ Channel Blockers				0.917 (0.803–1.047)
COX-2 Inhibitors				1.081 (0.984–1.186)
Glucocorticoids				1.036 (0.966–1.111)
Leukotriene Modifiers				1.060 (0.968–1.161)
Loop Diuretics				1.108***(1.030–1.192)
NSAIDs				1.089*** (1.021–1.161)
Statins				0.989 (0.922–1.061)
Thiazide Diuretics				0.979 (0.915–1.049)
Xanthine Derivatives				1.136** (1.003–1.286)

Notes: The primary exposure variable is *number of 30-day equivalent claims* of β2-agonist. Statistical significance is defined at the *10% level, **5% level, and ***1% level

Our comprehensive model (specification 4) further adjusts for smoking history, severity of asthma and COPD, and use of other drugs. The OR is 0.986 (95% CI, 0.977–0.995; P = 0.002). This indicates that an additional 30-day β2-agonist claim per year is associated with a 0.13% lower risk (in relative terms) of developing Parkinson’s disease. The ORs for other model variables are consistent with expectations. For example, age (OR = 1.296; 95% CI, 1.154–1.455; P<0.001), male sex (OR = 1.646; 95% CI, 1.488–1.822; P<0.001), and Alzheimer’s disease & related disorders (OR = 2.115; 95% CI, 1.967–2.273; P<0.001) were associated with increased incidence of PD, whereas acute myocardial infarction (OR = 0.768; 95% CI, 0.676–0.873; P<0.001) and tobacco use (OR = 0.900; 95% CI, 0.841–0.963; P<0.001) were negatively associated with PD incidence.

**Fig 1:**
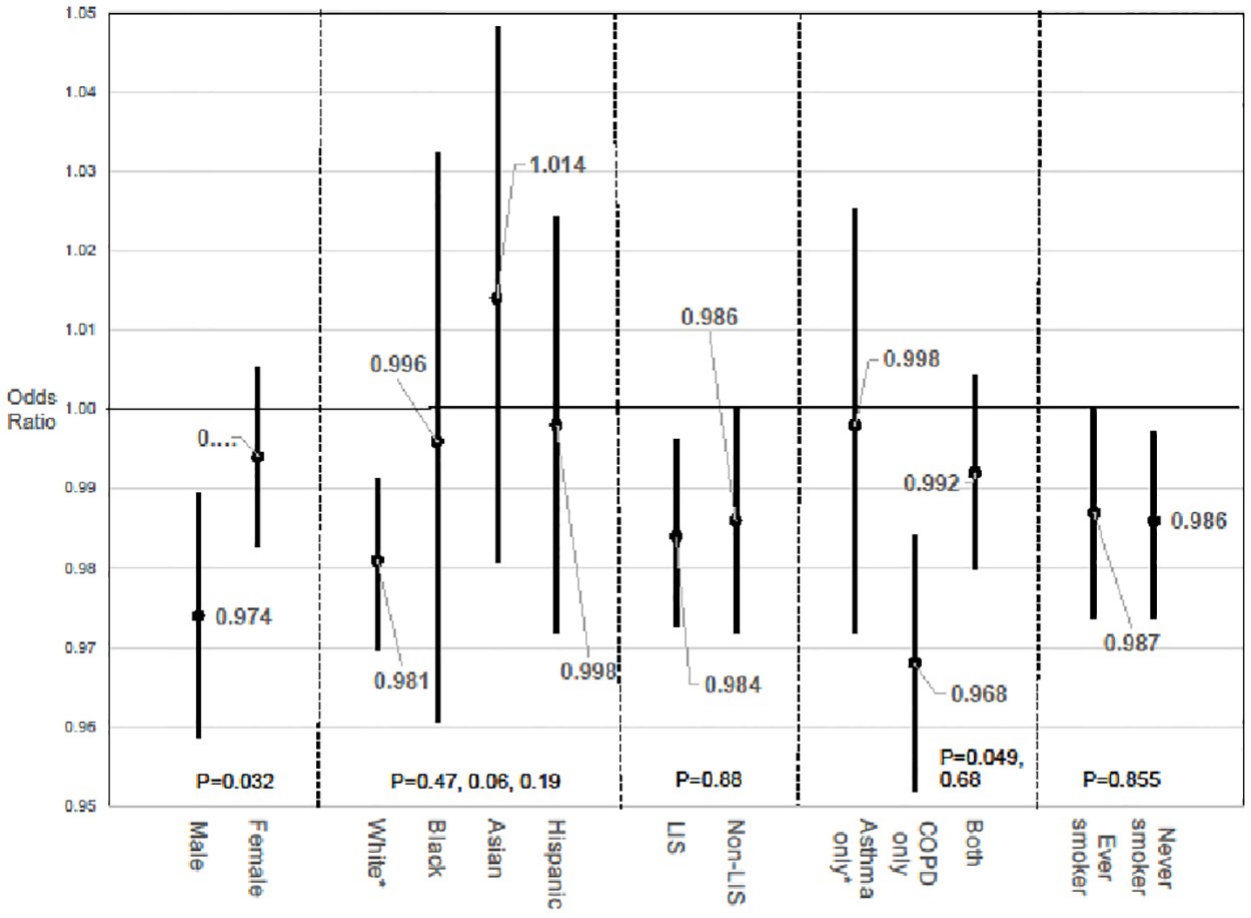
Incidence of Parkinson’s disease associated with β2-agonist use (30-day claims) by subgroup. P values at bottom are for within-group comparisons. For groups with more than two categories, P values are in relation to reference groups denoted by * in axis labels.

With full adjustment, Fig 1 plots ORs and confidence intervals of beta-agonist use on PD onset by subgroups: sex, race or ethnicity, Low Income Subsidy (LIS) status, disease status, and smoking status. In a test for equality of coefficients, chi-squared analysis showed the association between beta-agonist use and reduced PD incidence to be strongest among individuals who were male (OR = 0.974; 95% CI 0.959–0.989; p = 0.032 for the difference with females) or had a diagnosis of only COPD (p = 0.049 for the difference with those with only a diagnosis of asthma) at the 5% level. Race and ethnicity, income status, and smoking status did not appear to have a differential association between beta-agonist use and PD onset. Though the association for Asian ethnicity was insignificant (OR = 1.014, p = 0.41), the OR trended toward an increased likelihood of development of PD compared to individuals of White race (p = 0.059).

Results from the Cox model are reported in Table 4. The hazard ratio for β2-agonist use compared to no use is 1.656 (p<0.001). The hazard ratio for the interaction between β2-agonist use and time (months since initiation) is 0.983 (p<0.001). Together, these results imply an elevated risk of PD incidence immediately after β2-agonist initiation (hazard ratio of 1.656, p<0.001). By the end of follow-up (48 months), this elevated risk of PD onset diminishes such that disease incidence was less likely to be associated with beta-agonist users compared to non-users (hazard ratio of 0.719, p<0.001). When stratified by time since β2-agonist initiation, PD onset was associated with recent β2-agonist use prior to PD diagnosis, whereas reduced PD onset was associated with a longer duration of β2-agonist use.

**Table 4:** Cox analysis of β2-agonist use (30-day claims).

Variable	Hazard ratio (95% CI)
# of 30-day claims	1.656 (1.636–1.676)
# of claims, interacted with time (months)	0.983 (0.982–0.984)
Age	0.996 (0.989–1.002)
Male	1.629 (1.445–1.837)
Race, Hispanic	0.796 (0.697–0.908)
Race, Black	0.726 (0.621–0.849)
Race, Asian	1.053 (0.885–1.254)
Race, Other	1.119 (0.837–1.494)
*Comorbidities*: Acute myocardial infarction	0.744 (0.639–0.866)
Alzheimer’s Disease and Related Disorders or Senile Dementia	2.039 (1.875–2.217)
Anemia	1.106 (1.008–1.213)
Asthma	0.988 (0.901–1.085)
Atrial fibrillation	1.042 (0.943–1.151)
Benign Prostatic Hyperplasia	1.039 (0.922–1.172)
Cancer, Breast	0.960 (0.827–1.114)
Cancer, Colorectal	1.097 (0.925–1.300)
Cancer, Endometrial	1.000 (0.701–1.427)
Cancer, Lung	1.165 (0.920–1.474)
Cancer, Prostate	1.019 (0.878–1.181)
Cataracts	1.065 (0.982–1.155)
Chronic Kidney Disease	1.064 (0.944–1.200)
Chronic Obstructive Pulmonary Disease	1.050 (0.965–1.144)
Congestive Heart Failure	1.077 (0.997–1.164)
Depression	1.160 (1.068–1.260)
Diabetes Mellitus	0.920 (0.850–0.996)
Glaucoma	0.949 (0.814–1.108)
Hip fracture	1.286 (1.167–1.417)
Hyperlipidemia	0.983 (0.868–1.114)
Hypertension	1.041 (0.886–1.222)
Hypothyroidism	0.991 (0.915–1.074)
Ischemic Heart Disease	1.580 (1.463–1.707)
Osteoporosis	1.160 (1.068–1.260)
Rheumatoid arthritis	1.106 (1.008–1.213)
Stroke/Transient Ischemic Attack	1.300 (1.200–1.410)
Ever Smoker	0.906 (0.836–0.981)
Asthma Exacerbations	1.241 (1.157–1.330)
COPD Exacerbations	0.913 (0.858–0.972)
*Other Drug Use (%)*: ACE inhibitors	0.959 (0.887–1.036)
Anticholinergics	1.050 (0.946–1.166)
ARBs	0.972 (0.893–1.058)
Beta Blockers (Any, Selective)	1.011 (0.937–1.092)
Beta Blockers (Any, Non-Selective)	1.051 (0.948–1.166)
Ca^2+^ Channel Blockers	0.860 (0.735–1.006)
COX-2 Inhibitors	0.880 (0.783–0.988)
Glucocorticoids	1.365 (1.261–1.478)
Leukotriene Modifiers	1.075 (0.962–1.201)
Loop Diuretics	1.177 (1.083–1.280)
NSAIDs	1.096 (1.018–1.180)
Statins	0.933 (0.859–1.014)
Thiazide Diuretics	0.966 (0.891–1.048)
Xanthine Derivatives	0.768 (0.647–0.912)

## Discussion

This study examined the potential association between the use of β2-agonists in patients with asthma, COPD, and/or bronchiectasis and the incidence of Parkinson’s disease. Experimental studies have shown that the accumulation of the α-synuclein protein is linked to the development of Parkinson’s disease [8–10]. More recently, it was shown that activation of *B*_*2*_ adrenergic receptors modulates *SNCA* expression leading to a decrease in α-synuclein production, thereby playing a potential protective role in the development of Parkinson’s disease [13]. Currently, the evidence on the link between the use of β2-agonists and development of PD is inconclusive. Our study does find reverse causality bias to contribute to the documented association between B2AR use and PD onset. Nevertheless, after addressing reverse causality, our findings suggest that the use of β2-agonists in individuals with asthma, COPD, and/or bronchiectasis may reduce the incidence of Parkinson’s disease.

In observational studies such as this one, a cohort design (where feasible) can more effectively capture disease incidence and how various risk / protective factors modulate the development of the disease-state than alternative designs (such as cross sectional or case-control studies) [30]. Another advantage of the retrospective cohort design with a large sample is mitigation of sampling bias [31]. Additionally, the ability to recruit a large number of individuals into our sample allowed for subpopulation analyses, which is beneficial in examining differential effects of β2-agonists across subgroups.

In the subpopulation analysis, we conducted independent analyses followed by tests of equality of coefficients. As seen in Fig 1, significant differences in coefficients at the 5% level were seen between males and females as well as individuals with a diagnosis of COPD only compared to a diagnosis of asthma only. Individuals of White race compared to individuals of Asian ethnicity also appeared to exhibit a reduced association in onset of Parkinson’s disease following β2-agonist use (at the 10% significance level).

Gender bias has been documented in both diagnosis and treatment of asthma and COPD [32, 33]. More specifically, there are gender differences in the use of inhaled pharmacotherapies for patients with asthma, COPD, or asthma-COPD overlap. Females are found to have higher overall prescription rates of inhaled pharmacotherapies and were more likely to receive short-acting beta-agonists compared to males, however, males were more likely to be prescribed long-acting beta agonists compared to females [34]. The gender gap, particularly as it relates to beta-agonist use in the treatment of chronic obstructive lung diseases, could indeed influence the conferred benefit of beta-agonist use against PD onset.

We also found that β2-agonist users with COPD saw a larger reduction in OR compared to users with only asthma or both asthma and COPD. Though medication adherence is poor for maintenance therapies in the treatment of chronic respiratory illnesses, reports from the literature show that adherence may be higher in individuals with COPD compared to those with asthma. Better medication adherence is a plausible reason that may explain the stronger protective finding of β2-agonist use in the COPD subgroup [35].

Furthermore, disparities across race and ethnicity have also been documented in respiratory illness management. There is a reduced utilization of healthcare services and treatments among ethnic minority groups, which may explain why White individuals experienced a greater reduction in PD incidence following beta-agonist use [36–38].

Two recent studies have raised concerns about the possibility of reverse causality between β2-agonist use and PD incidence [15, 26]. We addressed these concerns with a Cox analysis of time-to-incidence that allowed β2-agonist use to vary between medication initiation and disease onset. We found a significantly elevated risk of Parkinson’s disease incidence immediately after β2-agonist initiation, consistent with reverse causality bias in which early symptoms of the disease might have influenced medication administration. However, this elevated disease incidence risk not only decreased with time since initiation, but we also found that users had a significantly lower risk of onset compared to non-users by the end of follow-up period, a finding consistent with a true protective effect from β2-agonist use.

### Limitations

Finding an inverse association between the use of beta blockers and PD onset may have provided additional evidence for the link between β2AR modulation and PD risk. Though we did find that beta blocker use trended in the direction of increasing PD incidence, our results were not significant. It is important to note, however, that in patients with asthma or COPD, administration of beta blockers comes at the expense of bronchodilation. Thus, our sample is not ideal for this issue and future studies may use better-suited patient samples to analyze the association between beta blocker use and PD incidence.

Another limitation of this study stems from a lack of clinical details in the observational data, which would include pertinent information on pulmonary function test results (FEV1 and FVC). This prevents us from directly assessing COPD and asthma severity, which have been linked to PD onset [16]. Nonetheless, we were able to proxy for asthma and COPD severity by identifying individuals who had frequent use of short-acting β2-agonists and controlling for number of asthma/COPD exacerbations by using number of inpatient and skilled-nursing facility stays.

Our use of claims data did not include information on quantity or duration of smoking and may have limited sensitivity in smoking history [39]. While imperfect, our measure of smoking history appears to have a substantial degree of reliability. We measured tobacco exposure via cessation claims made in Medicare parts A/B and deterrent claims made in Part D. In our sample, 36.1% of people had a measured smoking history, compared to 54.3% of older Americans in a recent nationally representative survey [40].

Given the protective nature of tobacco use on PD incidence as well as a positive association with β2-agonist use, we would expect that improper control for smoking use (underestimation) would overstate the true reduction in PD incidence due to β2-agonist use. Adding a smoking measure into this model would then be expected to weaken the association between β2-agonist use and PD incidence. In our analysis, we found that the association between β2-agonist use and PD incidence was not affected when adding the smoking variable into a model that previously did not include smoking history. This suggests that the effect of β2-agonist usage on PD incidence in our model is not confounded by smoking history.

While our analysis adjusted for a wide range of demographic and health-related factors, certain risks for PD, such as genetic makeup and environmental hazards, were unavailable, which could have resulted in confounding in this observational study. Lastly, our study sample is restricted to beneficiaries in Medicare FFS plans, as we do not observe claims records for Medicare Advantage enrollees. Including Medicare Advantage enrollees would have increased sample size and added power to our subpopulation analyses.

## Conclusion

Our study has shown a protective association between β2-agonist use and the onset of Parkinson’s disease. We did not find the use of beta blockers to impact incidence of Parkinson’s disease, but the study population is not ideal for this specific query. Subpopulation analysis shows a more pronounced reduction in PD incidence for individuals who are White, male, or have a diagnosis of COPD only. Analysis of time-varying covariates suggests that reverse causality accounts for PD incidence early in the study period but does not account for the effect that persists throughout the study timeline. These findings support the possibility that use of β2-agonists as maintenance therapy in patients with chronic obstructive respiratory illness may reduce the risk of PD onset.

To strengthen the evidence base, investigation into the documented association between β2-agonist use and Parkinson’s disease incidence by means of clinical trials and across particular medications are warranted. In addition, future research can further elucidate the findings of our subgroup analyses by more precisely investigating the mechanisms by which disparities across sex and race and ethnicity affect the interplay between pharmacologic management of COPD and/or asthma and development of Parkinson’s disease.

## Supporting information

S1 File (DOCX)
