# Supplementary material for: Association between use of ß2-adrenergic receptor agonists and incidence of Parkinson’s disease: Retrospective cohort analysis
Source: PLoS One. 2022 Nov 28;17(11):e0276368. doi: 10.1371/journal.pone.0276368 (PMC9704661; doi:10.1371/journal.pone.0276368)
Supplement: S1 File — (DOCX) [file pone.0276368.s001.docx]

# Author contributions

HN, BZ, DG and JR made substantial contributions to the conception and design of this project. HN, BZ and JR assisted with data acquisition, analysis, and interpretation of data for this manuscript. HN and JR drafted the manuscript, and BZ and DG revised it critically for important intellectual content. All authors approved of the final version to be published. JR is the guarantor.

# Financial Disclosure Statement

Research reported in this publication was supported by a grant from the National Institute on Aging of the National Institutes of Health under Award number P30AG024968. This funding organization played no role in design and conduct of the study; collection, management, analysis, and interpretation of the data; preparation, review, or approval of the manuscript; or the decision to submit the manuscript for publication.

# Competing interests

HN, BZ and JR have no competing interests. During the past three years, DG has received research support, speaker fees, travel assistance, or consulting income from the following sources: Amgen, Blue Cross Blue Shield of Arizona, Bristol Myers Squibb, Cedars-Sinai Health System, Edwards Lifesciences, Gates Ventures, Genentech, Gilead Sciences, Johnson & Johnson, Kaiser Family Foundation, National Institutes of Health, Novartis, Pfizer, Roche, and Walgreens Boots Alliance. DG holds equity in EntityRisk. DG reports personal fees from Biogen and GRAIL as a scientific advisor. Until November 2019, DG served on the Scientific Advisory Board of ACADIA Pharmaceuticals. Until March 2020, DG served as a scientific advisor to Precision Medicine Group. This does not alter our adherence to PLOS ONE policies on sharing data and materials.

# Data Availability Statement

The primary data source for the project is Medicare Research Identifiable Files (RIFs) and are not publicly available. The Medicare beneficiary summary file, Part A/B/D claims file and Chronic Conditions file are proprietary data from Center for Medicare and Medicaid Services (CMS). The authors are unable to share the data set as RIFs contain beneficiary level protected health information (PHI). Requests for RIF data require a Data Use Agreement (DUA) and are reviewed by CMS’s Privacy Board to ensure that the beneficiary’s privacy is protected and only the minimum data necessary are requested and justified. It would be illegal and against ethical restrictions to make the data analyzed in this study publicly available. A researcher can request access to the same data and obtain their own DUA through the CMS Research Data Assistance Center (<https://www.resdac.org>). Assistance for accessing and using these data is made available by the Research Data Assistance Center (ResDAC). ResDAC is a consortium of faculty and staff from the University of Minnesota, Boston University, Dartmouth Medical School, and the Morehouse School of Medicine. ResDAC provides free assistance to academic and non-profit researchers interested in using Medicare, Medicaid, SCHIP, and Medicare Current Beneficiary Survey (MCBS) data for research. The drug information file are propriety data from First Databank (FDB) Medknowledge and can be requested by contacting FDB (<https://www.fdbhealth.com/solutions/medknowledge-drug-database>).
